# Characterizing carbapenemase-producing *Escherichia coli* isolates from Spain: high genetic heterogeneity and wide geographical spread

**DOI:** 10.3389/fcimb.2024.1390966

**Published:** 2024-05-16

**Authors:** Elias Dahdouh, Laro Gómez-Marcos, Javier E. Cañada-García, Eva Ramírez de Arellano, Aida Sánchez-García, Isabel Sánchez-Romero, Luis López-Urrutia, Pedro de la Iglesia, Alejandro Gonzalez-Praetorius, Jared Sotelo, Daniel Valle-Millares, Isabela Alonso-González, Verónica Bautista, Noelia Lara, Silvia García-Cobos, Emilia Cercenado, Belén Aracil, Jesús Oteo-Iglesias, María Pérez-Vázquez, Spanish Eco-Carba Study Group, Verónica Casquero

**Affiliations:** ^1^ Laboratorio de Referencia e Investigación en Resistencia a Antibióticos e Infecciones relacionadas con la Asistencia Sanitaria, Centro Nacional de Microbiología, Instituto de Salud Carlos III, Madrid, Spain; ^2^ Centro de Investigacíon Biomédica en En Red de Enfermedades Infecciosas (CIBERINFEC), Instituto de Salud Carlos III, Madrid, Spain; ^3^ Servicio de Microbiología, URSalud UTE, Hospital Infanta Sofía, San Sebastián de los Reyes, Madrid, Spain; ^4^ Servicio de Microbiología, Hospital Puerta de Hierro, Madrid, Spain; ^5^ Servicio de Microbiología, Hospital Río Hortega, Valladolid, Spain; ^6^ Servicio de Microbiología, Hospital de Cabueñes, Asturias, Spain; ^7^ Servicio de Microbiología, Hospital Universitario de Guadalajara, Guadalajara, Spain; ^8^ Servicio de Microbiología, Hospital Universitario Gregorio Marañón, Madrid, Spain; ^9^ Centro de Investigacíon Biomédica en En Red (CIBER) de Enfermedades Respiratorias (CIBERES), Instituto de Salud Carlos III, Madrid, Spain

**Keywords:** carbapenemases, Escherichia coli, antibiotic resistance, virulence factor genes, whole-genome sequencing, sequence type, plasmids

## Abstract

**Introduction:**

Carbapenemase-Producing *Escherichia coli* (CP-Eco) isolates, though less prevalent than other CP-Enterobacterales, have the capacity to rapidly disseminate antibiotic resistance genes (ARGs) and cause serious difficult-to-treat infections. The aim of this study is phenotypically and genotypically characterizing CP-Eco isolates collected from Spain to better understand their resistance mechanisms and population structure.

**Methods:**

Ninety representative isolates received from 2015 to 2020 from 25 provinces and 59 hospitals Spanish hospitals were included. Antibiotic susceptibility was determined according to EUCAST guidelines and whole-genome sequencing was performed. Antibiotic resistance and virulence-associated genes, phylogeny and population structure, and carbapenemase genes-carrying plasmids were analyzed.

**Results and discussion:**

The 90 CP-Eco isolates were highly polyclonal, where the most prevalent was ST131, detected in 14 (15.6%) of the isolates. The carbapenemase genes detected were *bla*_OXA-48_ (45.6%), *bla*_VIM-1_ (23.3%), *bla*_NDM-1_ (7.8%), *bla*_KPC-3_ (6.7%), and *bla*_NDM-5_ (6.7%). Forty (44.4%) were resistant to 6 or more antibiotic groups and the most active antibiotics were colistin (98.9%), plazomicin (92.2%) and cefiderocol (92.2%). Four of the seven cefiderocol-resistant isolates belonged to ST167 and six harbored *bla*_NDM_. Five of the plazomicin-resistant isolates harbored *rmt*. IncL plasmids were the most frequent (45.7%) and eight of these harbored *bla*_VIM-1_. *bla*_OXA-48_ was found in IncF plasmids in eight isolates. Metallo-β-lactamases were more frequent in isolates with resistance to six or more antibiotic groups, with their genes often present on the same plasmid/integron. ST131 isolates were associated with *sat* and *pap* virulence genes. This study highlights the genetic versatility of CP-Eco and its potential to disseminate ARGs and cause community and nosocomial infections.

## 1 Introduction

Antibiotic-resistant *Escherichia coli* is one of the principal pathogens that can cause community-acquired and nosocomial infections with increased morbidity and mortality rates (Hu et al., 2022). They are considered as one of the principal causes of Urinary Tract Infections (UTIs), bacteremia, and Intra-Abdominal Infections (IAI) (Balasubramanian et al., 2023). *E. coli* have a heightened ability to acquire Antibiotic-Resistance Genes (ARGs), such as the *bla*_CTX-M-15_ Extended Spectrum β-Lactamase (ESBL), and rapidly disseminate them throughout the community (González et al., 2020). Carbapenemase-producing *Escherichia coli* (CP-Eco), though not as frequently isolated in the clinical setting as compared to other CP-producing Enterobacterales (such as *Klebsiella pneumoniae* and *Enterobacter cloacae* complex), is especially worrying. This is because they are increasing in prevalence (Cañada-García et al., 2022), creating a concern that they would be able to spread carbapenemase genes in the community in a similar manner as it was observed for ESBLs (González et al., 2020). Moreover, these isolates are typically resistant to several other antibiotics, making their related infections difficult to treat (Boutzoukas et al., 2023).

All main carbapenemase families have been detected in CP-Eco (Grundmann et al., 2017), in addition to a wide range of virulence determinants that negatively affect clinical outcome (Čurová et al., 2020). All this led to the declaration by the World Health Organization that CP-Eco are a critical priority issue (Tacconelli et al., 2018). Globally, antibiotic-resistant *E. coli* has the highest incidence of hospital-acquired infections in high-to-mid income countries, causing three to twenty-five million infections per year (Balasubramanian et al., 2023). In Europe, the median number of infections caused by CP-Eco was 2,619 in 2015, with a median of 141 attributable deaths (Cassini et al., 2019). In Spain, the incidence of CP-Eco has evolved from isolated cases in 2013 (Oteo et al., 2015), to being detected in 10 different Spanish provinces in 2019 (Cañada-García et al., 2022).

Given the importance and the widespread dissemination of CP-Eco, the aim of this study is the phenotypic and genotypic characterization of a set of such isolates obtained from different Spanish regions over a period of five years.

## 2 Materials and methods

### 2.1 Study design and bacterial collection

As part of the ongoing Antibiotic Resistance Surveillance Program, the Spanish National Center for Microbiology (CNM) receives clinical carbapenem-resistant E. coli isolates for molecular characterization from Spanish hospitals. Participation in this program is voluntary and the criteria to send the isolates to the CNM is having a Minimum Inhibitory Concentration (MIC) to meropenem of over 0.12 mg/L (Giske et al., 2017).

Of the total CP-Eco isolates received in this Surveillance Program between January 2015 and December 2020, a subset was selected for further analysis according to the following criteria: i) one isolate for each type of carbapenemase per year was included from each of the collaborating hospitals, ii) in the event that there was more than one isolate for each carbapenemase type in the same year, the selection was prioritized based on the type of infection in which they were involved according to the following order: 1) invasive infections 2) other infections 3) colonizations; iii) in all cases, the first isolate received per year was selected. These selection criteria allowed for a temporal-spatial representative sample of the different types of carbapenemases circulating in *E. coli* in Spain. Not all hospitals had isolates from all the five years, and from some hospitals more than one isolate per year was included since they produced different carbapenemase types. The information sent along with the isolate from the participating hospitals included collection date, age and sex of the patients, sample type, whether the isolate was a colonizer or causing infections (as determined by the attending microbiologist), and isolate origin [hospital, healthcare center, or community setting; *i.e.* where the infection is contracted without interaction with healthcare settings during the last 48 hours (Plachouras et al., 2018)].

### 2.2 DNA extraction and identification of carbapenemase genes

All the carbapenem-resistant *E. coli* isolates received at the CNM from 2015 to 2020 were cultured on MacConkey agar (Becton Dickinson Microbiology Systems, Cockeysville, MD, USA) and incubated overnight at 37°C. DNA was extracted using the QIAamp^®^ DNA Mini Kit (QIAGEN, Hilden, Germany) according to the manufacturer’s instructions. Real-time PCR assays targeting the carbapenemase gene families *bla*_OXA-48_, *bla*_KPC_, *bla*_VIM_, *bla*_IMP_ and *bla*_NDM_ were performed (Ortega et al., 2016). All the isolates were stored at -80°C until used.

### 2.3 Antibiotic susceptibility testing

Antibiotic Susceptibility Testing (AST) was determined for the selected isolates with broth microdilution using the YDKMGN Sensititre™ Gram Negative panels (Thermo Fisher, Waltham, MA, USA) (International Organization for Standardization (ISO), 2006). E-tests using meropenem/vaborbactam and imipenem/relebactam and disc diffusion assays for cefiderocol (Liofilchem, Roseto degli Abruzzi, Italy) were performed and interpreted according to the EUCAST guidelines (European Society of Clinical Microbiology and Infectious Diseases (EUCAST), 2022). Additionally, E-tests using plazomicin (Liofilchem, Roseto degli Abruzzi, Italy) and fosfomycin (bioMérieux, Marcy l’Etoile, France) strips were performed and interpreted according to an FDA-approved breakpoint of susceptibility of ≤2mg/L for plazomycin (Cañada-García et al., 2022), and according to the EUCAST cutoff values for urinary tract infections (UTI) for fosfomycin due to lack of clinical breakpoints for other types of samples (European Society of Clinical Microbiology and Infectious Diseases (EUCAST), 2022), respectively.

Resistance to different antibiotic families was analyzed considering 14 categories: nine categories according to the classification proposed by Magiorakos et al. (Magiorakos et al., 2012) (extended spectrum cephalosporins, monobactams, carbapenems, aminoglycosides, fluoroquinolones, fosphonic acid derivatives, folic pathway inhibitors, glycylcyclines and polymyxins); and 5 additional categories: ceftazidime/avibactam (extended spectrum cephalosporin + diazabicycloctane inhibitor), imipenem/relebactam (carbapenem + diazabicycloctane inhibitor), meropenem/vaborbactam (carbapenem + boronic acid inhibitor), cefiderocol (siderophore cephalosporin) and plazomicin (extended spectrum aminoglycoside).

### 2.4 Whole-genome sequencing and read assembly

DNA was extracted from the selected isolates as described in section 2.2. Paired-end (2x150) libraries were then prepared using the Nextera DNA Flex Library Preparation Kit and sequenced using Illumina NextSeq 550 (Illumina Inc., San Diego, CA, United States) according to the manufacturer’s instructions. Quality of the reads was assessed using FASTQC (version 0.11.9), followed by *de novo* assembly using Unicycler (version 0.4.8) (Wick et al., 2017). The quality of the assembly was assessed using QUAST (version 5.2.0) and annotation was performed using Prokka (version 1.14.6) (Seemann, 2014). The obtained assemblies were deposited in the European Nucleotide Archive under the accession number PRJEB70795.

### 2.5 Phylogenetic analyses and diversity

Ridom SeqSphere+ (version 8.3.1; Ridom, Münsten, Germany) was used to perform a core-genome Multi-Locus Sequence-Typing analysis (cgMLST) using a built-in scheme for *E. coli* containing 2,515 core genes, and to construct a minimum spanning tree based on allelic differences. ARIBA (version 2.6.2) (Hunt et al., 2017) was used to determine STs in accordance with the University of Warwick scheme. Diversity of the samples was calculated using a Simple Diversity Index [SDI (Gastmeier et al., 2006)]. Analysis of the *fim*H gene was done to subclassify ST131isolates using the FimTyper tool [CGE server; https://cge.cbs.dtu.dk; (accessed 23/02/2024)].

### 2.6 Antibiotic resistance genes, virulence-associated genes, and plasmids

ARGs were analyzed by ARIBA (version 2.14.6) (Hunt et al., 2017) using the CARD database (https://card.mcmaster.ca; (accessed 23/02/2024)) and ResFinder (CGE server) with ID thresholds of 100% for β-lactamase variants and 98% for other resistance genes. In isolates resistant to cefiderocol, the sequences of the genes encoding PBP3 and the siderophore CirA, previously related to resistance to this antibiotic (Wang et al., 2022), were analyzed with ARIBA.

The VirulenceFinder tool (CGE server) (accessed 23/02/2024) was used to detect virulence-associated genes (five toxin-coding genes, four adhesin-coding genes, two siderophore-coding genes, one outer membrane protein-coding gene and one invasive protein-coding gene). PlasmidID [https://github.com/BU-ISCIII/plasmidID; (accessed 23/02/2024)] was used to map the reads against a curated plasmid database, perform *de novo* plasmid assemblies, and determine the presence of resistance and replicon genes (Pérez-Vazquez et al., 2019).

### 2.7 Statistical analyses

The differences in prevalence of the ARGs, virulence-related genes, STs, and sample characteristics were evaluated using the Fisher’s exact test using GraphPad Prism (version 3.02; GraphPad Software, Inc., San Diego, CA, USA). *P* values of less than 0.05 were considered statistically significant.

### 2.8 Data availability

All the data generated for this study is available in the manuscript and its related **Supplementary Material**. The sequenced raw reads have been deposited in the open-access European Nucleotide Archive database under the accession number PRJEB70795.

## 3 Results

### 3.1 Characteristics of all the CP-Eco isolates received from 2015 to 2020

From January 2015 until December 2020, a total of 593 CP-*E. coli* isolates were confirmed as carbapenems producers by the Spanish CNM Reference Lab after screening for MIC to meropenem of over 0.12 mg/L by the collaborating hospitals. **Supplementary Table S1** shows the characteristics and origin of the isolates, as well as the information received from the participating hospitals (total and per year). The isolates were received from 25 Spanish provinces and 59 hospitals. Two-hundred-ninety-nine (50.4%) of the isolates were from females and the average patient age was 77.8 years. Three-hundred-one (50.8%) isolates were colonizers, and the rest were causing infections (24 were unspecified). The most common infection was UTI (175; 29.5%), followed by wound/ulcer infections (32; 5.4%), bacteremia (25; 4.2%), and IAI (17; 2.9%). Four-hundred-forty-three (74.7%) were healthcare-associated cases, 391 from hospitals and 52 from other healthcare centers, and 126 (21.2%) were from community settings; 24 were unspecified.

Using real-time PCR carbapenemase genes were detected in all 593 isolates where *bla*_OXA-48-like_ was detected in 485 (81.8%), *bla*_VIM-like_ in 71 (12%), *bla*_NDM-like_ in 17 (2.9%), and *bla*_KPC-like_ in 17 (2.9%) isolates. Three isolates harbored two carbapenemases, one of each of the following combinations *bla*_OXA-48_ and *bla*_KPC_, *bla*_OXA-48_ and *bla*_NDM_, and *bla*_VIM_ and *bla*_KPC_. In the community setting, the most frequent carbapenemase was *bla*_OXA-48-family_, detected in 109 (86.5%) isolates. The relative frequency of these genes per year was maintained throughout the study period and there was no statistically relevant correlation between the types of carbapenemase gene, sample type, location of isolation, infection, colonization, and type of carbapenemase harbored by the isolates (*P* > 0.05).

### 3.2 Characteristics and population structure of the 90 selected CP-Eco isolates

Based on the criteria set in section 2.1, 90 isolates from 59 hospitals across 25 Spanish provinces were selected. **Supplementary Table S2** lists all the characteristics of these isolates. Sixty (66.6%) isolates were implicated in clinical infections, as determined by the doctor who treated the patient, and mainly were obtained from urine (27; 45%), blood (15; 25%) and abscesses and wounds (12; 20%). The remaining 30 (33.3%) isolates were considered colonizing and came mainly from rectal swabs and feces (26; 86.7%)

Forty-two (46.7%) of the isolates were obtained from the hospital setting, the majority of which harbored *bla*_OXA-48_ (24; 57.1%), followed by *bla*_VIM-1_ (8; 19%), *bla*_NDM_ (3 *bla*_NDM-1_ and 3 *bla*_NDM-5_; 14.3% in total), *bla*_KPC_ (1 *bla*_KPC-2_ and 1 *bla*_KPC-3_; 4.8% in total), *bla*_OXA-162_ (1; 2.4%), and both *bla*_KPC-3_ and *bla*_OXA-48_ (1; 2.4%). Twelve isolates were obtained from healthcare centers where 3 harbored *bla*_OXA-48_, 3 *bla*_VIM-1_, 2 *bla*_NDM_ (1 *bla*_NDM-1_ and 1 *bla*_NDM-7_), 2 *bla*_KPC_ (1 *bla*_KPC-2_ and 1 *bla*_KPC-3_), 1 (8.3%) *bla*_OXA-162_, and 1 both *bla*_KPC-3_ and *bla*_OXA-48_. From the community setting, 18 (20%) of the isolates were collected where 8 had *bla*_OXA-48_, 4 *bla*_VIM-1_, 3 *bla*_NDM_ (1 *bla*_NDM-1_ and 2 *bla*_NDM-5_), and 3 *bla*_KPC_ (2 *bla*_KPC-2_ and 1 *bla*_KPC-3_). The origin of 18 isolates could not be determined.

The carbapenemase genes detected among the infecting isolates were *bla*_OXA-48_ (30; 50%), *bla*_VIM-1_ (13; 21.7%), *bla*_NDM_ (5 *bla*_NDM-1_ and 3 *bla*_NDM-5_; 13.3% in total), *bla*_KPC_ (3 *bla*_KPC-2_ and 3 *bla*_KPC-3_; 10% in total), one *bla*_OXA-162_ (1; 1.7%), and both *bla*_KPC_ and *bla*_OXA-48_ (2; 3.3%). Among the colonizing isolates, the carbapenemases genes observed were *bla*_OXA-48_ (11; 36.7%), *bla*_VIM-1_ (8; 26.7%), *bla*_NDM_ (6; 20%) (3 *bla*_NDM-5_, 2 *bla*_NDM-1_, and 1 *bla*_NDM-7_), *bla*_KPC_ (4; 13.3%) (3 *bla*_KPC-2_ and 1 *bla*_KPC-3_), and *bla*_OXA-162_ (1; 3.3%). No statistically significant associations were found between the sample type, location of isolation, infection, colonization, and type of carbapenemase harbored by the isolates (*P* > 0.05)

**Figure 1:**
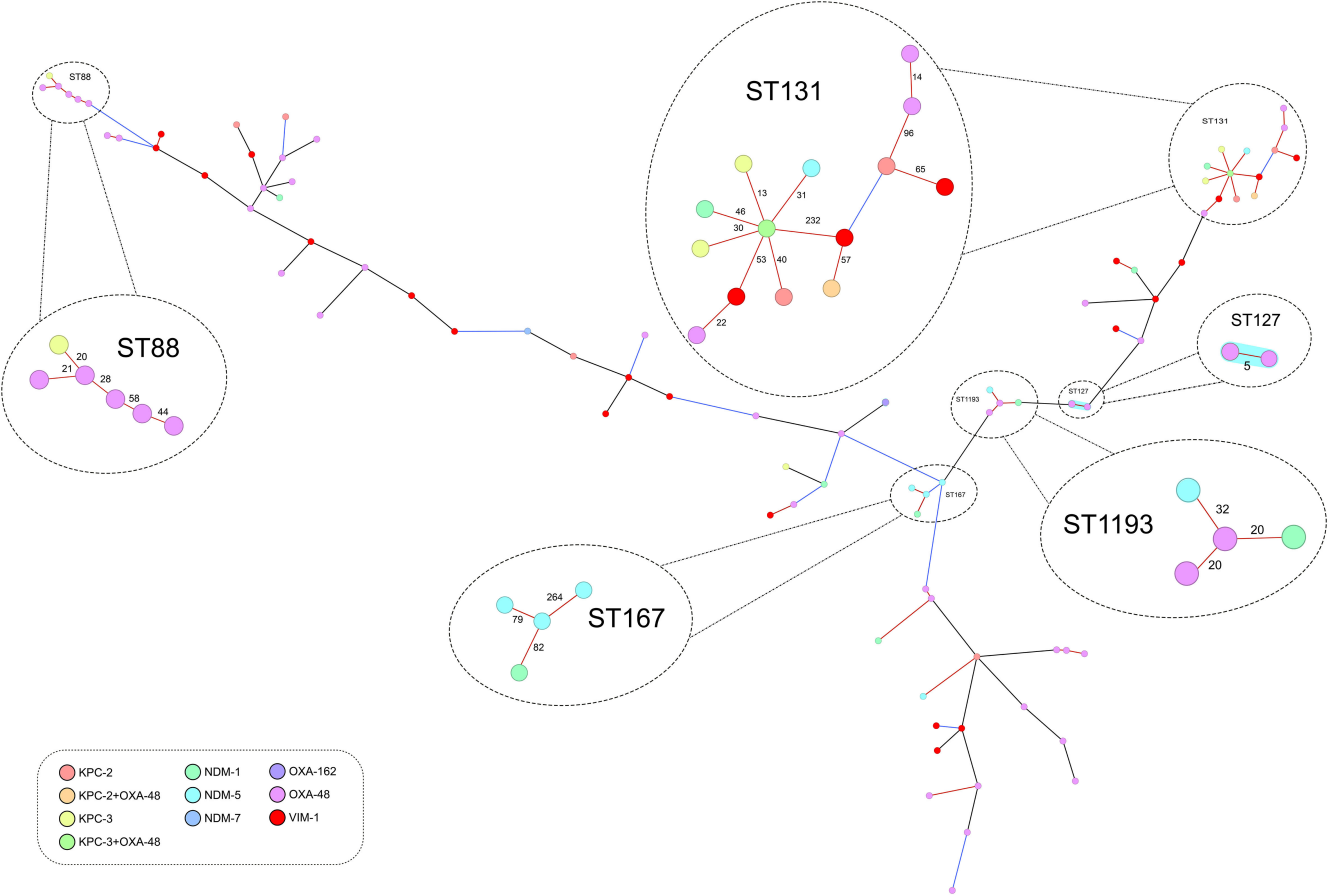
Minimum spanning tree showing the population structure of the 90 isolates analyzed through core-genome multi-locus sequence typing (scheme of 2,515 genes). The distance between the genes are not up to scale, black lines represent differences higher than 50% of the alleles considered in the scheme (>1257 alleles), blue lines differences between 10-50% (252 to 1257) and red lines differences lower than 10% (<252). Sequence types with four or more isolates are circled and amplified. The blue shading highlights the only group (containing two ST127 isolates) that have a genetic distance of ≤ 5 alleles (this group has also been amplified).

MLST analyses showed that the 90 isolates belonged to 49 different STs (SDI=54.4) and only ST131 (14 isolates) and ST88 (6 isolates) were detected in more than five isolates. Population diversity was similar for isolates harboring *bla*_OXA-48_ (SDI=65.1) and *bla*_NDM_ (SDI=64.3), and was lower than those harboring *bla*_KPC_ (SDI=71.0) and *bla*_VIM_ (SDI=81.0). Ridom SeqSphere+ was used to construct a maximum-likelihood phylogenetic tree using the gene-by-gene approach and allelic distance from cgMLST. **Figure 1** shows a great genetic diversity of the CP-Eco studied alone and in relation to the types of carbapenemases they had. The average allelic distances in pairwise comparison of all the CP-Eco isolates was 2,120 ± 555, and only four main clusters with ≥ 4 isolates were identified. The first cluster included 14 (15.6%) CP-Eco isolates that belonged to ST131, had an average of 105 allelic differences (range:13-660), were collected from 7 provinces, and all 4 carbapenemase gene families were detected in them (including two with both *bla*_OXA-48_ and *bla*_KPC-like_). The second-largest cluster was formed by 6 (6.7%) isolates belonging to ST88, had an average of 34 allelic differences (range:20-58), were collected from 5 provinces and 5 isolates harbored *bla*_OXA-48_. Cluster 3 contained 4 (4.4%) isolates belonging to ST167 that all harbored *bla*_NDM_, were collected from 3 provinces and had an average of 142 allelic differences (range:79-264). Cluster 4 contained 4 (4.4%) isolates pertaining to ST1193 collected from 2 provinces with an average allelic difference of 24 (range:20-32); two of them harbored *bla*_OXA-48_ and the other two *bla*_NDM_. The rest of the isolates were grouped into STs with a frequency of 3 or less isolates. Applying a ≤ 5 allele differences threshold to identify possible transmission events, only one cluster of two OXA-48-producing ST127 isolates were detected.

Further typing of the *fim*H gene was conducted on the predominant ST131. Among the 14 ST131 isolates, 7 (50%) had *fim*H30 indicative of belonging to clade C, 4 (28.6%) had *fim*H41 (clade A), 1 (7.1%) had *fim*H22 (clade B), 1 (7.1%) had *fim*H298 (subtype of clade B), and 1 (7.1%) had *fim*H1196 (no specific clade). *bla*_CTX-M-15_ and *bla*_KPC_ genes were more frequent in ST131 clade C isolates (71.4% and 57.1%, respectively) than in non-clade C isolates (14.3% and 28.6%, respectively) but these differences were not statistically significant (*P* > 0.05).

### 3.3 Characterization of the acquired antibiotic resistance genes

**Table 1** shows the detected ARGs where an average of 5.6 ARGs were detected (range:1–13; excluding chromosomal genes and mutations). *bla*_OXA-48-like_ was detected in 43 (47.8%) isolates, *bla*_VIM-1_ in 21 (23.3%), *bla*_NDM-like_ in 14 (15.6%), *bla*_KPC-like_ in 10 (11.1%), and both *bla*_OXA-48_ and *bla*_KPC-like_ in 2 (2.2%) isolates. Isolates harboring *bla*_VIM-1_ (7.2; range:3-11) and *bla*_NDM-like_ (6.7; range:1-12) had more ARGs than those isolates harboring *bla*_OXA-48_ (5; range:1-13) and *bla*_KPC-like_ (4.6; range:1-12). Twelve isolates harbored only one ARG, six of them had *bla*_OXA-48-like_, five had *bla*_KPC-like_, and one had *bla*_VIM-1_. Isolates belonging to ST88 had an average of 9.8 ARGs (range:7-13). *bla*_OXA-48_ was most frequently encountered in ST88 isolates (5; 11.6%), *bla*_NDM-like_ in ST167 (4; 28.6%), *bla*_KPC-like_ in ST131 (6; 54.6%) and *bla*_VIM-like_ in ST131 isolates (3; 14.3%). *bla*_OXA-162_ was detected in 2 isolates belonging to ST5968.

**Table 1:** Genes and mutations associated with antibiotic resistance detected through whole-genome sequencing.

Carbapenemases	n (%)	ESBLS	n (%)	Other β-Lactamases	n (%)
***bla*_OXA-48_**	41 (45.6%)	*bla*_CTX-M-15_	13 (14.4%)	*bla*_OXA-1_	17 (18.9%)
***bla*_VIM-1_**	21 (23.3%)	*bla*_CTX-M-14_	6 (6.7%)	*bla*_CMY_	4 (4.4%)
***bla*_NDM-1_**	7 (7.8%)	*bla*_SHV-12_	3 (3.3%)	*bla*_TEM-30_ & *bla*_OXA-1_	1 (1.1%)
***bla*_KPC-2_**	6 (6.7%)	*bla*_CTX-M-24_	2 (2.2%)	*bla*_TEM-34_	1 (1.1%)
***bla*_NDM-5_**	6 (6.7%)	Others^2^	3 (3.3%)	*bla*_DHA-1_	1 (1.1%)
***bla*_KPC-3_**	4 (4.4%)				
***bla*_OXA-162_**	2 (2.2%)				
**Others^1^**	3 (3.3%)				
Chloramphenicol	n (%)	Fosfomycin	n (%)	Tetracycline	n (%)
***catA*(*1*&*2*) + *catB*(*2*&*3*)**	11 (12.2%)	*ptsI*_V25I + *uhpT*_E350Q	14 (15.6%)	*tet(A)*	15 (16.7%)
***catB*(*2*&*3*)**	8 (8.9%)	*uhpT*_E350Q	11 (12.2%)	*tet(B)*	12 (13.3%)
***cmlA1***	5 (5.6%)	Others^3^	2 (2.2%)	*tet(A)* + *tet(M)*	1 (1.1%)
***floR***	4 (4.4%)				
***catA1***	3 (3.3%)				
***catA1* + *floR***	3 (3.3%)				
TMX	n (%)	Quinlones (chr.)	n (%)	Quinlones (acq.)	n (%)
***sul*(*1*,*2*&*3*)**	43 (47.8%)	*gyrA* + *parC* + *parE*	37 (41.1%)	*qnrS*(*1*,*2*,&*11*)	15 (16.7%)
***dfrA* ^4^**	29 (32.2%)	*gyrA* + *parC*	8 (8.9%)	*qnrB*(*2*,*4*&*19*)	5 (5.6%)
***dfrB1***	7 (7.8%)	*gyrA*	8 (8.9%)	Others^6^	3 (3.3%)
***dfrA*(*5*&*17*) + *dfrB1***	2 (2.2%)	Others^5^	3 (3.3%)		
AMEs	n (%)	Amino Methyl.	n (%)	Rifamycin	n (%)
***aad(A1,A2&A16)***	52 (57.8%)	*rmtC*	3 (3.3%)	*arr-3*	1 (1.1%)
***aac(6’)*(-lb&*-Ib-cr*)**	30 (33.3%)	*rmtB1*	1 (1.1%)	*rpoB_*V146F	1 (1.1%)
***aac(3)*(*-IId,-IIe*&*-IVa*)**	13 (14.4%)	*rmtF1*	1 (1.1%)		
***ant(2’’)*-*Ia***	3 (3.3%)				
***aph(3’)*(-*VI*&-*XV*)**	2 (2.2%)				

^1^One of each: bla_KPC-2_ & bla_OXA-48_, bla_KPC-3_ & bla_OXA-48_ and bla_NDM-7_.

^2^One of each: bla_CTX-M-27_, bla_CTX-M-9_ and bla_PER-2_.

^3^One of each: fosA3 and fosA7.5.

^4^Variants detected are 1, 5, 8, 12, 14, 17, 27 and 36.

^5^One of each: parC, parE and gyrA & pare.

^6^One of each: qnrA1 & qnrS2, qnrB1 & qnrS1, and qnrVC1. “ESBL” stands for “Extended-Spectrum Beta-Lactamases”, “Chr. mutations” refers to “chromosomal”, “Acq.” refers to “Acquired genes”, “TMX” refers to Trimethoprim/Sulfamethoxazole, “Amino Methyl.” refers to “*16S rRNA* Methylases that confer resistance to aminoglycosides”, and “AMEs” refer to “Aminoglycoside Modifying Enzymes”.

Acquired ESBL genes were detected in 27 (30%) isolates where *bla*_CTX-M-15_ was predominant (n=13; 48.1%). *bla*_PER-2_ was detected in a *bla*_NDM-1_-harbouring isolate belonging to ST12. Regarding acquired AmpC genes, *bla*_CMY_ was detected in four isolates, three of them belonged to ST167 and all four harbored *bla*_NDM-like_ (**Supplementary Table S2**).

The non-β-lactamase acquired ARGs present in at least 25% of the CP-Eco isolates were the sulfonamide-resistance-encoding *sul* (n=64, 71.1%; mainly *sul*1); trimethoprim-resistance-encoding *dfr*A (n=56, 62.2%; mainly *dfr*A17); tetracycline-resistance-encoding *tet* (n=38, 42.2%; mainly *tet*A); aminoglycoside-resistance-encoding *aac(6’)* (n=31, 34.4%; mainly *aac(6’)-Ib*) and *aad*A (n = 52, 57.8%; mainly *aad*A1); chloramphenicol-resistance-encoding *cat* (n=25, 27.8%; mainly *cat*A1); and quinolone-resistance-encoding *qnr* (n=23, 25.6%; mainly *qnr*S1) (**Table 1**; **Supplementary Table S2**). Twenty-five isolates had chromosomal mutations associated with resistance to fosfomycin and 2 additional isolates harbored the *fos*A7.5 and *fos*A3 acquired genes. These genes were located in chromosomal and plasmid contigs, respectively. The aminoglycoside methyltransferase gene *rmt* was detected in five isolates belonging to different STs, where 4 harbored *bla*_NDM-1_ and 1 *bla*_NDM-5_.

### 3.4 Characterization of the plasmids carrying the carbapenem resistance genes

The median size of the detected plasmids was 63,598 bp with a median coverage of 98% and a median similarity of 100% with the respective entries in Genbank. IncL plasmids were the most frequently detected (n=42; 46.7%). Thirty-two of these harboured *bla*_OXA-48_, eight harboured *bla*_VIM-1_, and two harboured *bla*_OXA-162_. The second most frequent plasmid type was IncFII detected in 12 (13.3%) isolates, eight of which harbored *bla*_OXA-48_ and four harbored *bla*_NDM_. Five of the IncF-harboring isolates also belonged to clade C of ST131. IncN was detected in five (5.3%) isolates where four harbored *bla*_VIM-1_ and one harbored *bla*_KPC-2_. IncP6 was detected in four isolates harboring *bla*_KPC-2_, and IncFIB was detected in four of the five isolates harboring *bla*_KPC-3_. Other plasmid types were detected with a frequency of two or less and it was not possible to fully reconstruct the carbapenemase-harboring plasmids in 20 isolates (**Supplementary Table S2**).

No other ARG was detected on the same plasmid harboring *bla*_OXA-48-like_ nor for those harboring *bla*_KPC-like_, except for one isolate harboring *bla*_KPC-2_ that also harbored a class 1 integron with the sequence *Int1*-*aac(6’)-Ib-arr3-dfrA27-aadA16-qacE*Δ*1-sul1*. *bla*_VIM-1_ in IncL plasmids was found in a class 1 integron with the sequence *Int1-bla_VIM-1_-aac(6’)-Ib-dfrB1-aadA1-catB2-qacE*Δ*1-sul1*. In four other isolates, IncN plasmids harbored class 1 integron with *bla*_VIM-1_ but with a different combination of genes. Six isolates harbored *bla*_NDM-5_ (n=4), *bla*_NDM-1_ (n=1) or *bla*_NDM-7_ (n=1) in complex class 1 integrons with different combinations of aminoglycoside-resistance genes, including *rmt*C. Another isolate had *bla*_NDM-1_ and *rmt*C on the same plasmid, but they did not form part of an integron (**Supplementary Table S2**).

### 3.5 Antibiotic resistance profiles

**Table 2** shows the AST results that were analyzed according to the categories described in section 2.3. All isolates had MICs>0.12mg/L to meropenem, harbored at least one gene of carbapenem resistance, and were resistant to at least one carbapenem (**Table 2**). The two isolates producing two carbapenemases presented relatively low MICs of 4 mg/L to imipenem (both) and 4 and 1 mg/L to meropenem (each). Antibiotics with *in-vitro* activity against more that 90% of the isolates were colistin, plazomicin, cefiderocol, and meropenem/vaborbactam. The *mcr* genes and known mutations in the PhoPQ and PmrAB encoding genes related to colistin resistance were not detected in the colistin-resistant isolate.

**Table 2:** Antibiotic susceptibility profiles of the 90 selected isolates determined through broth microdilution and interpreted according to the EUCAST guidelines.

Antibiotic Category	Antibiotic	Range S – R (mg/L)	MIC_50_ (mg/L)	MIC_90_ (mg/L)	S	SIE	R
**Carbapenems**	**Ertapenem**	0.25 – >2	2	>2	16 (17.8%)	N/A	74 (82.2%)
	**Imipenem**	≤0.5 – >16	2	16	53 (58.9%)	14 (15.6%)	23 (25.6%)
	**Meropenem**	≤0.12 – >16	1	16	68 (75.6%)	10 (11.1%)	12 (13.3%)
**Carbapenem + Diazabicyclooctane**	**Imipenem + relebactam**	≤2 – >2	1	8	61 (67.8%)	N/A	29 (32.2%)
**Carbapenem + Boronate**	**Meropenem + vaborbactam**	≤8 – >8	0.5	16	82 (91.1%)	N/A	8 (8.9%)
**Extended Spectrum Cephalosporins**	**Ceftazidime**	≤0.5 – >16	>16	>16	24 (26.7%)	13 (14.4%)	53 (58.9%)
	**Cefotaxime**	≤0.5 – >8	>8	>8	10 (11.1%)	13 (14.4%)	67 (74.4%)
**Cefalosporin + Diazabicyclooctane**	**Cefftazidime + avibactam**	≤0.5/4 – >16/4	1/4	>16/4	52 (57.8%)	N/A	38 (42.2%)
**Cephalosporin + Siderophore**	**Cefiderocol**	≤2 – >2	0.025	1	83 (92.2%)	N/A	7 (7.8%)
**Monobactam**	**Aztreonam**	≤0.5 – >32	≤0.5	>32	47 (52.2%)	5 (5.6%)	38 (42.2%)
**Aminoglycosydes**	**Gentamicin**	≤0.5 – >8	2	>8	51 (56.7%)	N/A	39 (43.3%)
	**Amikacin**	≤4 – >32	≤4	32	74 (82.2%)	N/A	16 (17.8%)
	**Tobramicin**	≤1 – >8	8	>8	44 (48.9%)	N/A	46 (51.1%)
**Extended- Spectrum Aminoglycoside**	**Plazomicin**	≤2 – >2	1.5	2	83 (92.2%)	N/A	7 (7.8%)
**Folic Pathway Inhibitors**	**Trimethoprim + sulfamethoxazole**	≤1/19 – >8/152	>8/152	>8/152	35 (38.9%)	3 (3.3%)	52 (57.8%)
**Phosphonic Acid Derivative**	**Fosfomycin**	≤8 – >8	2	4	82 (91.1%)	N/A	8 (8.9%)
**Fluoroquinolones**	**Ciprofloxacin**	≤0.06 – >2	>2	>2	27 (30.0%)	3 (3.3%)	60 (66.7%)
**Glycylcylins**	**Tigecyclin**	≤0.25 – 4	0.5	1	71 (78.9%)	N/A	19 (21.1%)
**Polimixins**	**Colistin**	≤0.25 – >8	1	1	89 (98.9%)	N/A	1 (1.1%)

“MIC” stands for Minimum Inhibitory Concentration, “N/A” stands for “Not Applicable”, “R” stands for “Resistant”, “S” stands for “Susceptible”, and “SIE” stands for “Susceptible, Increased Exposure”.

Forty isolates (44.4%) were resistant to more than six out of the 14 antibiotic categories considered. These isolates harbored *bla*_VIM-1_ (n=15; 37.5%), *bla*_NDM-like_ (n=13; 32.5%), *bla*_OXA-48-like_ (n=7; 17.5%), *bla*_KPC-like_ (n=4; 10%), and both *bla*_KPC-3_ and *bla*_OXA-48_ (n=1; 2.4%). Nine isolates (7 NDM-producers, 1 VIM-producer, and 1 OXA-48-producer) were only susceptible to three or four antibiotic categories. The most active agents against these isolates were colistin (9/9) and tigecycline (6/9).

**Table 3** shows the antibiotic resistance rates of the CP-Eco isolates, grouped by the type of carbapenemase gene harbored. Isolates harboring *bla*_OXA-48_ had lower resistance rates towards meropenem and imipenem as compared to non-*bla*_OXA-48_-harboring isolates (4.7% and 7% versus 21.3% and 46.2%, respectively; *P* = 0.0003). OXA-48 and ESBL co-producers (n=15) were significantly more resistant to ceftazidime (46.7%), cefotaxime (100%), and aztreonam (100%) than OXA-48 producers without ESBL (n=26, with 3.8%, 15.4%, and 3.8% resistance rates, respectively (*P*<0.05)). No other β-lactamases combinations could be statistically studied in relation to antibiotic resistance profiles due to the very low number of isolates for each combination. All the isolates resistant to imipenem/relebactam (n=29), ceftazidime/avibactam (n=26) or meropenem/vaborbactam (n=8) were metallo-β-lactamase-(MBL)-producers, except for one OXA-48-producer that was resistant to meropenem/vaborbactam and imipenem/relebactam, one OXA-48-producer resistant to imipenem/relebactam and ceftazidime/avibactam, and two isolates resistant to ceftazidime/avibactam producers of KPC-3/OXA-48 and OXA-48 carbepenemases, respectively. Three of these isolates presented insertions in the *omp*C gene in comparison with ertapenem-resistant isolates from this same study but susceptible to the rest of the carbapenems and to 3rd generation cephalosporins and their combinations. Insertions detected were E87_N88insD in one OXA-48 producer, and T225_A226insGLNGYG in one OXA-48- and one KPC-3/OXA-48-producing isolates. However, these insertions do not generate a premature stop codon. *omp*F mutations were not detected.

**Table 3:** Number and percentages of the CP-Eco isolates that are resistant to the tested antibiotics grouped by the carbapenemase gene that they harboured.

		*bla*_OXA-48_ (n = 43)	*bla*_VIM_ (n = 21)	*bla*_NDM_ (n = 14)	*bla*_KPC_ (n = 12)
**Carbapenems**	**Ertapenem**	41 (95.3%)	8 (38.1%)	14 (100.0%)	11 (91.7%)
	**Imipenem**	3 (7.0%)	5 (23.8%)	13 (92.9%)	2 (16.7%)
	**Meropenem**	2 (4.7%)	1 (4.8%)	8 (57.1%)	1 (8.3%)
**Carbapenem + Diazabicyclooctane**	**Imipenem + relebactam**	2 (4.7%)	13 (61.9%)	14 (100.0%)	0 (0.0%)
**Carbapenem + Boronate**	**Meropenem + vaborbactam**	1 (2.3%)	0 (0%)	7 (50.0%)	0 (0.0%)
**Extended Spectrum Cephalosporins**	**Ceftazidime**	21 (48.8%)	21 (100.0%)	14 (100.0%)	11 (91.7%)
	**Cefotaxime**	8 (18.6%)	21 (100.0%)	14 (100.0%)	10 (83.3%)
**Cefalosporin + Diazabicyclooctane**	**Cefftazidime + avibactam**	2 (4.7%)	21 (100.0%)	14 (100.0%)	1 (8.3%)
**Cephalosporin + Siderophore**	**Cefiderocol**	0 (0%)	1 (4.8%)	6 (42.9%)	0 (0.0%)
**Monobactam**	**Aztreonam**	13 (30.2%)	2 (9.5%)	11 (78.6%)	12 (100.0%)
**Aminoglycosydes**	**Gentamicin**	9 (21.0%)	18 (85.7%)	7 (50.0%)	4 (33.3%)
	**Amikacin**	3 (7.0%)	4 (19.0%)	7 (50.0%)	1 (8.3%)
	**Tobramicin**	12 (27.9%)	21 (100.0%)	8 (57.1%)	4 (33.3%)
**Extended Spectrum Aminoglycoside**	**Plazomicin**	0 (0.0%)	1 (4.8%)	5 (35.7%)	0 (0.0%)
**Folic Pathway Inhibitors**	**Trimethoprim + sulfamethoxazole**	20 (46.5%)	15 (71.4%)	11 (78.6%)	6 (50.0%)
**Phosphonic Acid Derivative**	**Fosfomycin**	3 (7.0%)	3 (14.3%)	2 (14.3%)	0 (0.0%)
**Fluoroquinolones**	**Ciprofloxacin**	26 (60.5%)	13 (61.9%)	13 (92.9%)	8 (66.7%)
**Glycylcylins**	**Tigecyclin**	9 (20.9%)	4 (19.0%)	2 (14.3%)	4 (33.3%)
**Polimixins**	**Colistin**	1 (2.3%)	0 (0.0%)	0 (0.0%)	0 (0.0%)

Six of the seven isolates resistant to cefiderocol were NDM-producers (4 *bla*_NDM-5_ and 2 *bla*_NDM-1_) and one had *bla*_VIM-1_. Four NDM-producers belonged to ST167 and had an insertion in the *pbp*3 gene (Y334insYRIN), in addition to two other mutations in the same gene (E349K and I532L). Three of these isolates additionally had a frameshift mutation in the siderophore gene *cir*A (V89fs). Another NDM-producer (ST131) had a truncation in *cir*A (W243trunc), and the final NDM-producer belonged to ST405, had the Y334insYRIN insertion in the *pbp*3 gene, and a frameshift mutation in *cir*A (T508fs). The VIM-producer belonged to ST23 and had a frameshift mutation in *cir*A (T190fs). The median inhibition zone for NDM-producers was significantly lower compared to non-NDM-producers (20.5 versus 29.5; *P* = 0.00001).

There were six isolates that were resistant to all aminoglycosides, including plazomicin, and that carried *rmt* methyltransferases (3 *rmt*C, 1 *rmt*F1 and 1 *rmt*B1), with five of them additionally having *bla*_NDM-like_. All 16 amikacin-resistant isolates harbored *aac(6’)-Ib* and/or methyltransferases genes, while the 46 tobramycin-resistant isolates had *aac(3)-II*, *aac(6’)-Ib*, *ant(2’’)-Ia* or *rmt*B1 genes (**Supplementary Table S2**). Two NDM-producers were resistant to aztreonam with no other β-lactamase gene detected, while all other MBL-producers resistant to this antibiotic harbored ESBL and/or AmpC genes (**Supplementary Table S2**).

Finally, 82 isolates (91.1%) had fosfomycin MICs of ≤ 8 mg/L and were considered susceptible (using EUCAST criteria for UTIs). Of those, 21 isolates (25.6%) had chromosomal mutations associated with resistance to fosfomycin. Of the 8 isolates resistant to this antibiotic, two harbored *fos*A genes, three had the chromosomal mutations ptsI_V25I/uhpT_E350Q, one had the chromosomal mutation uhpT_E350Q, and in the last two no known genetic mutation or gene was identified. There was a significant correlation between harboring mutations or genes associated with fosfomycin resistance and phenotypic resistance to this antibiotic (*P* = 0.003).

### 3.6 Characterization of the virulence-associated genes

**Table 4** shows the detected virulence-associated genes (13 virulence genes considered), and the number of infection-causing isolates harboring them. The isolates had a median of two virulence-associated genes (range=0-9) where adhesin-coding genes were the most prevalent (68.9%). Thirty-eight isolates (42.2%) had four or more virulence-associated genes, 19 of which were causing infections. The most common infection in this group was UTI (9), followed by bacteremia (5), IAIs (2), wound infections (2), and abscess (1) (**Supplementary Table S2**). Isolates with 4 or more virulence-associated genes included three isolates belonging to ST131 and two belonging to ST88; and had similar averages of ARGs and susceptibility rates to the different antibiotic categories. Nineteen of these 34 isolates had *bla*_OXA-48_, 8 had *bla*_VIM-1_, 3 had *bla*_NDM-5_, 2 had *bla*_NDM-1_, 1 had *bla*_KPC-3_, and 1 had both *bla*_OXA-48_ and *bla*_KPC-3_. Of the isolates pertaining to ST131, all had *fim*H and over half of them harbored *pap* and *omp*A. Over half of those pertaining to ST88 harbored *fim*H, *iro*N, and *omp*A.

**Table 4:** Virulence-associated genes detected in the all 90 carbapenemase-producing *E. coli* isolates, in addition to the two most frequent sequence types and the number of isolates harboring these genes that were causing infections.

Adhesin-Coding Genes						
	All Isolates (n = 90)		ST131 Isolates (n = 14)		ST88 Isolates (n =6)	
Gene	n (%)	Causing Infection	n (%)	Causing Infection	n (%)	Causing Infection
***fimH***	65 (72.2%)	44	14 (100%)	9	4 (66.7%)	3
***pap***	30 (33.7%)	20	9 (62.3%)	7	2 (33.3%)	2
***sfa***	27 (30.3%)	18	6 (42.9%)	5	2 (33.3%)	2
afa	7 (7.9%)	6	0 (0%)	0	2 (33.3%)	2
Toxin-Coding Genes						
	All Isolates (n = 90)		ST131 Isolates (n = 14)		ST88 Isolates (n =6)	
Gene	n (%)	Causing Infection	n (%)	Causing Infection	n (%)	Causing Infection
***sat***	13 (14.6%)	7	6 (42.9%)	5	0	0
***vat***	11 (12.4%)	7	0 (0%)	0	0	0
***hlyA***	9 (10.1%)	7	2 (14.3%)	1	0	0
***cnf1***	9 (10.1%)	6	3 (21.4%)	1	0	0
***pic***	2 (2.2%)	2	0 (0%)	0	0	0
Siderophore-Coding Genes						
	All Isolates (n = 90)		ST131 Isolates (n = 14)		ST88 Isolates (n =6)	
Gene	n (%)	Causing Infection	n (%)	Causing Infection	n (%)	Causing Infection
***iroN***	15 (16.9%)	14	0 (0%)	0	4 (66.7%)	4
***iutA***	1 (1.1%)	0	0 (0%)	0	0 (0%)	0
Outer Membrane Protein-Coding Genes						
	All Isolates (n = 90)		ST131 Isolates (n = 14)		ST88 Isolates (n =6)	
Gene	n (%)	Causing Infection	n (%)	Causing Infection	n (%)	Causing Infection
***ompA***	69 (77.5%)	47	10 (71.4%)	7	4 (66.7%)	4
Invasive Protein-Coding Genes						
	All Isolates (n = 90)		ST131 Isolates (n = 14)		ST88 Isolates (n =6)	
Gene	n (%)	Causing Infection	n (%)	Causing Infection	n (%)	Causing Infection
***ibeA***	4 (4.5%)	2	1 (7.1%)	0	0 (0%)	0

The adhesin-coding *pap* and the toxin-coding *sat* genes were found to be more frequent among isolates pertaining to ST131 (*P* = 0.02 and 0.0004, respectively). Particularly, five of the 9 isolates positive for *pap* and five out of the six positive for *sat* belonged to clade C. Additionally, the adhesion-coding *fimH* gene had almost double the frequency among isolates harboring *bla*_OXA-48_ as compared to isolates harboring other carbapenemase genes (*P* = 0.04). There were no statistically significant differences in the virulence-associated genes between bloodstream isolates and isolates from other sources.

## 4 Discussion

In this study, 90 representative CP-Eco isolates obtained from 25 Spanish provinces from 2015 to 2020 were characterized. The inclusion criteria were designed to achieve the greatest possible representation of geographic areas, clones, and carbapenemases, as well as to avoid overrepresentation due to outbreaks and/or endemics. They do not reflect the real prevalence of the carbapenemase genes; an approach to this more general view is provided in section 3.1, which mirrored the epidemiological situation of CP-Eco in Spain (Cañada-García et al., 2022). Notably, CP-Eco isolates were found in 25 Spanish provinces, and major STs, such as ST131, showed inter-provincial spread. The studied CP-Eco isolates were highly polyclonal, unlike other Enterobacterales, such as *Klebsiella pneumoniae* that tend to show less genetic diversity (Oteo et al., 2015; Lázaro-Perona et al., 2022).

The high-risk ST131 was the most prevalent in this collection; this ST also represented 36% of CP-Eco isolates from 16 countries between 2008 and 2013 (Patiño-Navarrete et al., 2020). In Spain, ST131 represented 16.5% of CP-Eco isolates collected between 2012-2014 (Ortega et al., 2016). Furthermore, isolates belonging to this ST harbored all the carbapenemase genes detected in this study, on different plasmids and in different combinations with other acquired ARGs. This demonstrates its potential to act as a vector for disseminating resistance genes in different scenarios. Half of the ST131 isolates caused infections, and all of them were community-acquired. Moreover, a significant association was found between ST131 and the virulence genes *sat* (toxin-coding) and *pap* (adhesin-coding), highlighting its potential to cause life-threatening community-acquired infections.

The ST167/NDM-5 clone, observed in this study, had been previously described (Poirel et al., 2022b) and had shown to spread rapidly across European borders (Linkevicius et al., 2023). These isolates were also resistant to cefiderocol and detected in three different provinces. Of note, 6/7 of the cefiderocol-resistant isolates were also NDM-producers, an association that has been previously drawn, especially among ST167 isolates having the same mutations in *pbp*3 as shown in our study and a premature stop codon in the siderophore gene *cir*A (Wang et al., 2022). Additionally, NDM-producers were found to have tighter inhibition zones towards cefiderocol compared to non-NDM-producers, in line with recent findings where NDM-producers had elevated MICs towards this antibiotic (Poirel et al., 2022a). This hints at an interplay between NDM and cefiderocol resistance that warrants further investigation.

Another finding in our study is the detection of a relatively high rate of MBL-producers in isolates recovered from the community setting. The high average age of these patients could suggest an origin in nursing homes that are known reservoirs of CP-producing bacteria (Palacios-Baena et al., 2016) and whose flow of admissions-discharges from and to the community is difficult to trace. Moreover, *bla*_VIM-1_ was detected in small IncL plasmids similar to the highly transmissible ones that carried *bla*_OXA-48_ (Boyd et al., 2022). This combination was detected in isolates belonging to different STs, as previously described in *K. pneumoniae* (Cañada-García et al., 2023), and was predominantly found in healthcare-associated settings. This raises concern regarding the fast dissemination of *bla*_VIM-1_, especially in Spain where the prevalence of VIM-producers is higher than other countries (Johnston et al., 2021; Cañada-García et al., 2022). Of note, *bla*_OXA-48_ was in IncFII plasmids in eight isolates, an observation that was reported in another study though *bla*_OXA-48_ was part of a different transposon (Moussa et al., 2020).

The CP-Eco isolates harboring *bla*_OXA-48_ were significantly more susceptible to imipenem and meropenem compared to CP-Eco isolates harboring other carbapenemases, in accordance with the known weaker hydrolytic activity of this enzyme towards carbapenems (López-Camacho et al., 2019). Moreover, all OXA-48-like-producers and all but one KPC-producer did not harbor any other ARG on their plasmids, unlike other Enterobacterales such as *K. pneumoniae* where it is typical to find other ARGs with *bla*_KPC_ (Cañada-García et al., 2022; Cañada-García et al., 2023). In contrast, 30-40% of MBL-producers were resistant to more than six antibiotic categories as defined in this study. All the “new” antibiotics and antibiotic/inhibitor combinations, except imipenem/relebactam, showed over 75% *in-vitro* activity towards the CP-Eco isolates. Resistance to imipenem/relebactam, meropenem/vaborbactam or ceftzidime/avibactam was mainly due to MBL production (Galani et al., 2019). Three of the only four isolates resistant to at least one of these combinations had insertions in the OmpC porin gene, in comparison with sequences from OXA-48-producing isolates of this study only resistant to ertapenem but susceptible to the rest of the carbapenems and 3rd generation cephalosporins. The presence of mutations in the main porins of *Enterobacterales* has previously been related to resistance to the new beta-lactamase inhibitors combinations (Navarro-Carrera et al., 2022). VIM-producers appeared to be more resistant towards imipenem/relebactam than imipenem alone, which has been previously reported in another study (Hernández-García et al., 2022). However, this was mainly caused by the EUCAST cutoff values (European Society of Clinical Microbiology and Infectious Diseases (EUCAST), 2022) used to determine susceptibility since six of the eight VIM-1 producing isolates resistant to imipenem/relebactam but susceptible to imipenem had MICs of 4 mg/L for both antibiotics. There were also seven NDM-producers that were susceptible to meropenem/vaborbactam according to the EUCAST guidelines. However, these isolates had elevated MICs, and most would have been considered resistant in case the CLSI guidelines were followed (Jean et al., 2019).

Six isolates were resistant to plazomicin, five of them harbored *bla*_NDM-like_ and were the only isolates in which *rmt* methyltransferases were detected. Furthermore, unlike other Enterobacterales in which *rmt* methyltransferase gene seems to disseminate via clonal expansion (Arca-Suárez et al., 2022), in our collection this gene was detected in isolates from different STs and were present in different plasmids.

Fosfomycin is one of the main antibiotics used to treat community-acquired uncomplicated cystitis caused by *E. coli* in Spain (Sojo-Dorado et al., 2022). In this study, most of the isolates that had mutations associated with resistance to this antibiotic harbored chromosomal mutations, and only two isolates had *fos* genes, similarly to what was previously described (Falagas et al., 2019). Moreover, the *fos*A7 genes detected in the chromosome of one of the isolates has also been described to be able to be present there (Pan et al., 2021). The correlation between resistance to fosfomycin and the presence of resistance mechanisms against this antibiotic must be taken with caution since cut-off points for UTI have been used (the only ones established by EUCAST), of which there were only 26 isolates in this collection. Moreover, although a good agreement was reported between using E-test strips and disk diffusion assays (Hirsch et al., 2015; Goer et al., 2022), the EUCAST does not explicitly recommend the use of E-tests for the determination of cutoff values for fosfomycin. Nevertheless, these isolates could be displaying a heterorresistant phenotype that appeared to be susceptible in our testing methods and criteria (Campos et al., 2020).

In conclusion, our data shows a wide range of genetically diverse CP-*E. coli* isolates present across several Spanish geographical areas, with a heterogeneous set of ARGs, plasmids, and virulence-associated genes. These findings highlight the potential of this bacteria to acquire and disseminate ARGs, and to cause life-threatening, difficult-to-treat infections in different settings, including the community. The spread of CP-Eco in the community, like what already happened with CTX-M-15-producing *E. coli*, would pose a threat of great impact to the general health of the population. The integration of WGS in the surveillance and diagnosis of CP-Eco, in line with ECDC recommendations, can help control and minimize this impact.

## Data availability statement

All the data generated for this study is available in the manuscript and its related **Supplementary Material**. The sequenced raw reads have been deposited in the open-access European Nucleotide Archive database under the accession number PRJEB70795.

## Author contributions

ED: Writing – review & editing, Writing – original draft, Validation, Methodology, Investigation, Data curation. LG-M: Writing – review & editing, Writing – original draft, Methodology, Investigation, Data curation. JC-G: Writing – review & editing, Writing – original draft, Visualization, Software, Investigation, Data curation. ER: Writing – review & editing, Writing – original draft, Investigation, Formal Analysis, Data curation. AS-G: Validation, Formal Analysis, Writing – review & editing, Writing – original draft, Resources. IS-R: Formal Analysis, Writing – review & editing, Writing – original draft, Validation, Resources. LL-U: Validation, Formal Analysis, Writing – review & editing, Writing – original draft, Resources. PI: Validation, Formal Analysis, Writing – review & editing, Writing – original draft, Resources. AG-P: Validation, Resources, Formal Analysis, Writing – review & editing, Writing – original draft. JS: Writing – review & editing, Writing – original draft, Methodology, Investigation. DV-M: Writing – review & editing, Writing – original draft, Software, Investigation, Data curation. IA-G: Writing – review & editing, Writing – original draft, Investigation, Data curation. VB: Writing – review & editing, Writing – original draft, Methodology, Investigation. NL: Writing – review & editing, Writing – original draft, Methodology, Investigation. SG-C: Writing – review & editing, Writing – original draft, Investigation, Formal Analysis, Data curation. EC: Validation, Writing – review & editing, Writing – original draft, Resources, Formal Analysis. BA: Writing – review & editing, Writing – original draft, Validation, Supervision, Resources, Project administration, Formal Analysis. JO-I: Writing – review & editing, Writing – original draft, Validation, Supervision, Resources, Project administration, Funding acquisition, Formal Analysis. MP-V: Writing – review & editing, Writing – original draft, Visualization, Supervision, Software, Resources, Formal Analysis.

## Other members of the Spanish Eco-Carba study group are:

Verónica Casquero and Olga Valiente (Centro Nacional de Microbiología, Instituto de Salud Carlos III, Madrid), Almudena Alhambra Mosquera (Hospital Universitario HM Montepríncipe, Madrid), Alia Eworo Ndongo (Hospital de El Escorial, Madrid), Susana Hernando Real (Hospital General de Segovia), Luis Moisés Ruiz-Velasco (Hospital Central De La Cruz Roja San José y Santa Adela, Madrid), José Leiva (Clínica Universitaria de Navarra), Nieves Balado (Hospital Xeral de Vigo, Pontevedra), Adriana Ortega (Fundación Hospital de Alcorcón, Madrid. CIBERINFEC), Mar Olga Pérez Moreno (Hospital de Tortosa Verge de La Cinta, Tortosa, Tarragona), Ana Bordes (Hospital De Gran Canaria Dr. Negrín, Las Palmas de Gran Canaria), Cristobal del Rosario Quintana (Hospital Universitario Insular de Gran Canaria), María Eugenia Portillo (Hospital Universitario de Navarra), Caridad Sainz de Baranda (Complejo Hospitalario Universitario de Albacete), Gloria Trujillo (Hospital San Joan de Deu (Althaia), Barcelona), Begoña Palop (Hospital Regional de Málaga), Carmen Aldea-Mansilla (Complejo Asistencial Universitario de Soria), Juan Cuadros (Hospital Universitario Príncipe de Asturias, Madrid), Yolanda Gil (Hospital de Móstoles, Madrid), Soledad Illescas Fernández-Bermejo (Hospital General Universitario de Ciudad Real), Ana Ramos (Hospital Universitario de Torrejón, Madrid), Salvador Giner (Hospital Universitario La Fe, Valencia), Antonio Casabella Pernas (Corporación Sanitaria Parc Taulí, Barcelona), M. Pilar Ortega Lafont (Complejo Asistencial Universitario de Burgos), María Huertas Vaquero (Hospital General La Mancha Centro, Ciudad Real), Isabel Antolín (Hospital de Medina del Campo, Valladolid), M^a^ de los Ángeles Pallarés (Hospital Montecelo, Pontevedra), Beatriz Iglesias (Hospital San Agustín, Asturias), Frederic Gómez-Bertomeu (Hospital Universitario Joan XXIII, Tarragona; CIBERINFEC), Ana Isabel López-Calleja (Hospital Universitario Miguel Servet, Zaragoza), Pilar Zamarrón (Hospital Virgen de La Salud, Toledo)
